# Study protocol for a randomized control trial investigating the effectiveness of a multifaceted mHealth approach on adherence to antihypertensive treatment among patients in Pakistan

**DOI:** 10.12669/pjms.41.1.9272

**Published:** 2025-01

**Authors:** Muhammad Arshed, Aidalina Binti Mahmud, Muhammad Farooq Umer, Fawad Mashhadi, Ayesha Babar Kawish

**Affiliations:** 1 Dr. Muhammad Arshed, Ph.D. University Institute of Public Health, Faculty of Allied Health Sciences, The University of Lahore, Pakistan; 2 Dr. Aidalina Binti Mahmud, Ph.D. Department of Community Health, Faculty of Medicine and Health Sciences, University Putra Malaysia; 3 Dr. Muhammad Farooq Umer, Ph.D. Department of Preventive Dental Sciences, College of Dentistry, King Faisal University, Hofuf, Al-Ahsa, Saudi Arabia; 4 Dr. Syed Fawad Mashhadi, Ph.D. Department of Community Medicine, Army Medical College, NUMS, Rawalpindi, Pakistan; 5 Dr. Ayesha Babar Kawish, MSPH Al-Shifa School of Public Health, Al-Shifa Trust, Rawalpindi, Pakistan

**Keywords:** Mobile Health, mHealth, Interventions, Medication adherence, Hypertension

## Abstract

**Background & Objectives:**

Poor medication adherence is an essential contributor to Pakistan’s high prevalence of uncontrolled hypertension. This study will be aimed to assess the efficacy of a one-of-a-kind developed intervention in improving medication adherence and treatment outcomes in hypertension patients.

**Methods:**

Twleve months duration long randomized controlled trial from January to December 2021 will be carried out at Shaikh Zayed Medical Complex (SZMC), Lahore. A total of 440 patients aged 18 years and older diagnosed with hypertension in the last month with non- adherence to antihypertensive therapy <80% of pills used in the last 30 days and have access to a smartphone will be randomized into either the intervention group (n=220) or the control group (n=220). For the intervention arm, a comprehensive intervention, the “Multi-Aid-Package,” consisting of seven items: written, voice, and graphics messages, animated video, educational material, and a 24/7 help service, has been designed. Standard care will be provided to the control group. The primary outcome will be improved adherence to antihypertensive medication, while the secondary outcome will be an alteration in systolic blood pressure (SBP). The analysis will be intention to treat.

**Conclusion:**

According to this study, if the multifunctional Multi-Aid-Package proves to be a useful mobile health tool for improving hypertension patients’ medication adherence, it will also significantly affect systolic blood pressure. In Pakistan and other comparable low- and middle-income countries LMICs, the Multi-Aid-Package ought to be taken into consideration as a means of enhancing adherence to medications among hypertension patients.

## INTRODUCTION

Hypertension is a growing global health problem in the 21st century. Globally, 1.13 billion people are hypertensive, and approximately 75% of people are in LMICs.^1^ About 9.4 million fatalities worldwide are attributed to hypertension, thus becoming a major cause of death.^2^ However, these fatalities are avoidable, as cardiovascular events and all-cause mortality can be decreased by reducing systolic blood pressure.^3^

Hypertension is diagnosed in 18.9% of adolescents 15 years of age or older.^4^ The prevalence rates for hypertension were reported as 58.1%.^5^ It has been noted that modifiable variables such as a sedentary lifestyle, inadequate food, and poor education may raise the chance of undetected hypertension.^6^ Poor medication adherence and factors affecting adherence should be taken into account.^7^,^8^ Poor blood pressure control results from non-adherence to medication with a reported prevalence of non-adherence was 37.7%.^9^ Non-adherence is linked to poorer blood pressure control and adverse outcomes.^10^

In LMICs, in particular, it has been discovered that using mHealth initiatives to increase drug adherence is found effective.^11^ mHealth refers to medical care delivered using cell phones, patient monitoring devices, personal digital assistants, and supplementary wireless technologies.^12^ Because of its low cost and ease of use, mHealth is an ideal tool for low-resource settings. Mobile devices, cellular communication technology, and an internet connection are needed. As stated by the Telecommunication Authority of Pakistan, 57% of the Pakistani population uses smartphones.^13^ While the evidence that mHealth can improve medication adherence is largely lacking where very few studies done in LMICs. Additionally, there is a need to better understand how these digital solutions can be seamlessly integrated into existing clinical workflows, as well as their impact on patient behavior and overall adherence. There is a gap in standardized measures for evaluating medication adherence in studies involving mHealth, making it difficult to compare results across different studies and populations.^11^,^14^ Recommendations have been made for more research in LMICs to establish a strong causal association between the effectiveness of m-health in improving medication adherence in patients with CVDs.^11^ For this purpose, More trials are required to incorporate more refined designs.^11^

Therefore, a cutting-edge model will be employed to evaluate its efficacy comparing with traditional approaches. According to our information, this research is the first of its kind a multi-model approach with all seven comprehensive components derived from the Health Belief Model (HBM) and Self-Determination Theory (SDT) related to professional guidelines, recommendations, and technology application theories, not being unitizing in such a way in preexisting literature.

### Research Objectives:

- To assess the impact of a multimodal mobile health intervention on medication adherence in individuals receiving antihypertensive therapy.
- To assess the impact of a multimodal mobile health intervention on SBP in individuals receiving antihypertensive therapy.

### Research hypothesis:

- There is no significant impact of a multimodal mobile health intervention on medication adherence in individuals receiving antihypertensive therapy.
- There is no significant impact of a multimodal mobile health intervention on systolic blood pressure in individuals receiving antihypertensive therapy.

## METHODS

**Fig.1:**
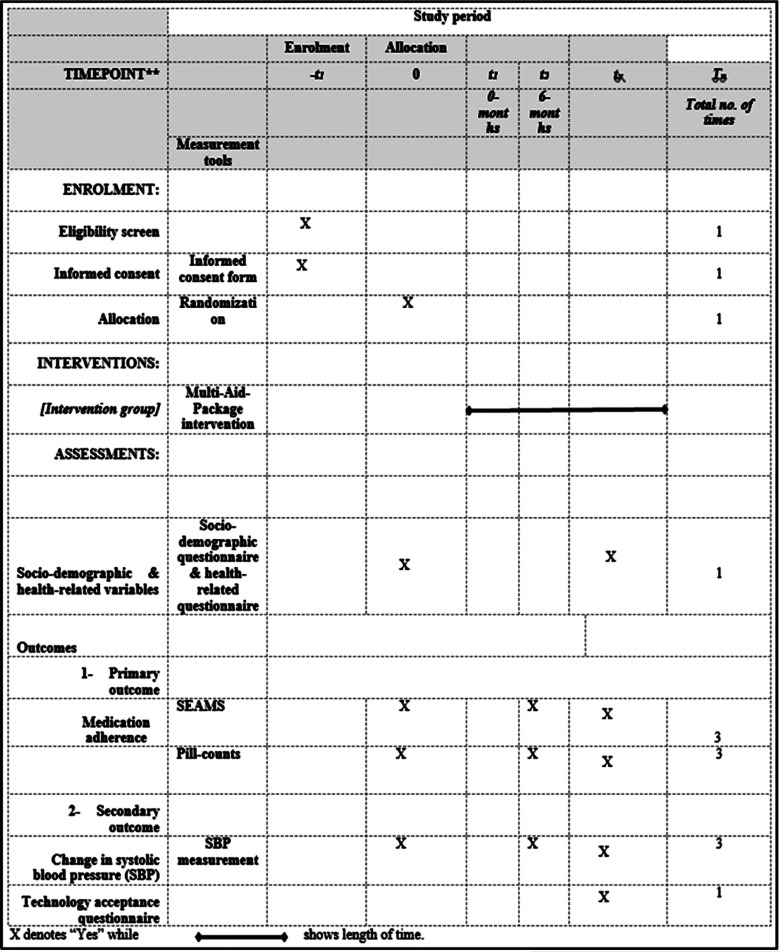
Timelines and schedule for the trial, t_x_: total follow-up time

This study is 12 months duration long overall from January to December 2021, including six months follow up period, two-arm, single-blinded, intention-to-treat randomized controlled trial. The evaluation will be performed at the pretest and posttest. This trial is designed in accordance with the SPIRIT 2013 guidelines (Fig.1).^15^

**Fig.2:**
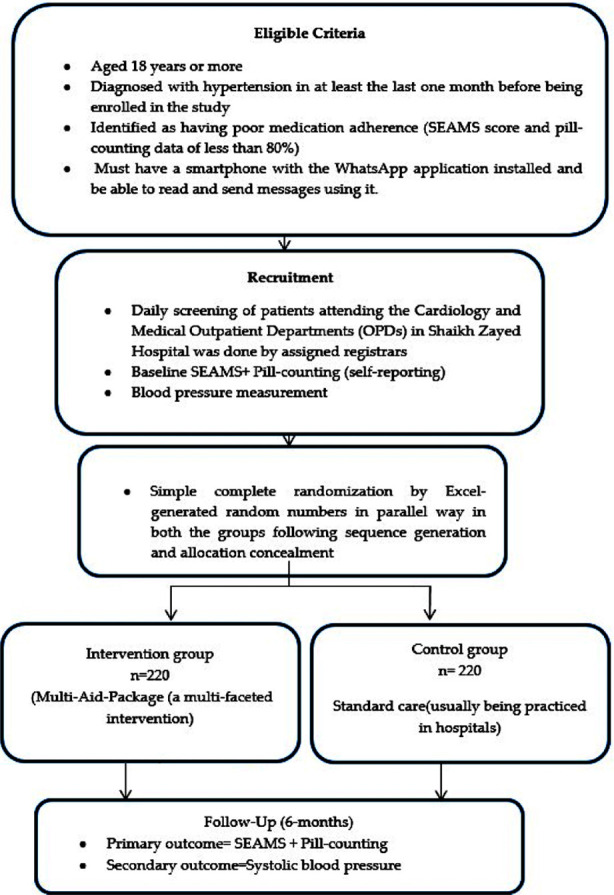
Algorithm for trial steps

### Study Setting:

The research will be conducted at Shaikh Zayed Medical Complex (SZMC), Lahore, a public tertiary care facility. Lahore is Punjab’s provincial capital.

### Study Participants:

The study participants will be hypertensive patients registered in SZMC’s Cardiology and Medical Outpatient Departments (OPDs).

### Ethical Considerations:

The University Putra Malaysia Ethical Committee on Human Research (JKEUPM-2020-391; May 19, 2022) and SZMC, IRB (Reference number: SZMC/IRB/163/2021; dated August 3, 2021) have accepted and approved the research protocol.

### Inclusion Criteria

- Aged 18 years or more.
- Hypertension diagnosed in at least the last month before enrolment in the study.
- They must be registered as hypertensive patients at a public tertiary care hospital’s cardiology and medical outpatient departments and have been prescribed antihypertensive medications.
- Identified as non-adherent to medication (SEAMS score and pill-counting data of less than 80%).
- Must be capable of reading and sending messages using a smartphone and WhatsApp.

### Exclusion Criteria

- Have travel plans in the following two months resulting in not having access to mobile signals.
- Patients who have had cancer in the past may need to adjust their medication.
- Patients with a hypertensive emergency reading of more than 220/120 mmHg
- Patients who report being pregnant, lactating, or three months postpartum

### Intervention Arm:

The focus of the intervention will be to increase the adherence of those in the intervention group taking treatment for hypertension. The contents of this module will be based on the HBM and SDT.

The intervention will be a seven-item, multifaceted educational support and inimitable module for reminders called the “Multi-Aid Package” that will be delivered using a mobile phone application (WhatsApp), in addition to the standard treatment (according to the hospital’s protocol).

The module will be comprised of daily audio and textual, graphics-based reminders (GBR), and, two times a week, graphics-based messages (GBM). Reminder text, voice, and graphics for medication intake are in Urdu. Examples of such statements will be “Good Morning; it is time for your medicine.” There will be a weekly animated video presentation containing educational material on hypertension and medical and lifestyle adaptations for good Health, which resultantly improves adherence. Based on the two well-known theories, Health believe model (HBM) and Self-determination theory (SDT), an experienced team will be involved in designing a graphic-based communications series and video that improves motivation and self-efficacy for antihypertensive treatment.

The participants can also access the “Hypertension at a Glance” module to obtain complete information about their diseases, such as their etiology, diagnosis, treatment, consequences, and complications. Multi-Ad Package has been designed as a more personalized and customized approach for patients. Each component addresses to provide a highly personalized strategy for a patient-centered intervention (Table-I).

**Table-I:** Contents of Multi-Aid-Package.

Multi-Aid-Package Multifaceted educational and reminder module		

No.	Content Description	Frequency
1	Written message	Daily
2	Voice message	Daily
3	Graphics-based reminder (GBR)	Daily
4	Graphics-based message (GBM)	Twice weekly
5	Hypertension at a glance	Optional
5.1	PowerPoint presentation	Optional
5.2	Literature support as the convenience of participant	Optional
6	Animated video	Weekly
7	Help provided by the doctor	Optional

### Control Arm:

Participants in the control group receive conventional care only. No intervention/ placebo will be provided to controls. There will be no way to keep them engaged except follow-ups.

### Outcome Measures

### Primary Outcome:

The change in adherence to antihypertensive medication will be the primary outcome. The primary endpoint will be assessed using the SEAMS and pill counts used over a certain period.^14^,^16^ The adherence to antihypertensive medication will be assessed at baseline and six months.

### Secondary Outcome:

The change in SBP will be the secondary outcome. Two readings will be taken, and the final measurement is the average of the two. Systolic blood pressure will be assessed at baseline and six months.

### Sample Size:

The sample size is calculated using the Lemeshow et al. 1990s formula: 
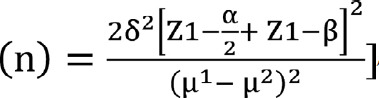
17 to detect between the two groups on the adherence change to the primary outcome measure. The adherence score in the intervention group is 7.4, and in the control, it is 7.0.^18^. The alpha level is set at 0.05, a confidence interval of 95%. The calculated sample size is 220 in each group and 440 overall after a 30% attrition rate.

### Randomization, Sequence generation, and Allocation concealment:

A simple, complete randomization technique will be used.^19^ Initially, the formula =ROUNDUP (RAND ()*440,0 will be used in Microsoft Excel to create a random sequence. Using their unique identification numbers, participants will be randomly assigned 1:1 into each group in a parallel manner. When the sealed opaque envelopes with the screened participants’ identification numbers are opened, it will be clear which group (control or intervention) they are assigned to. Every ongoing randomization phase will be carried out by a biostatistician.

### Blinding:

**Fig.3:**
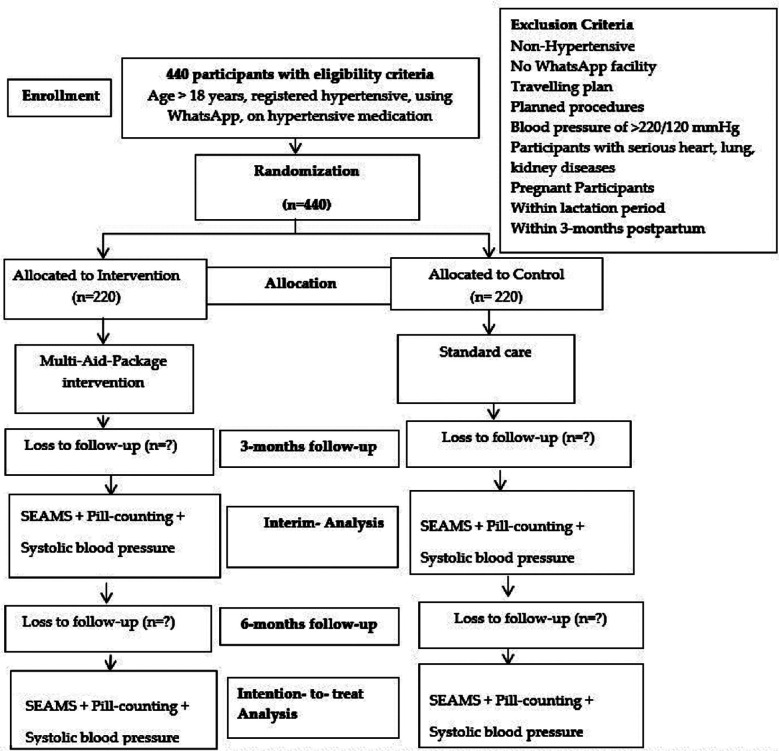
Flow Chart.

The data collectors and outcome evaluators will be unaware (blinded) of the group allocation. The flow chart (CONSORT) is provided in Fig.3.

### Data Collection:

A four section validated and pretested questionnaire in Urdu and English will be utilized. Section-A will involve the collection of data on pertaining to health and sociodemographics. Section B will be the the SEAMS-related scale.^20^ A 13 item measure called SEAMS is used to evaluate medication adherence.^20^ The scale’s scores go between 13 and 39. Greater levels of scores are correlated with better scores.

In Section-C, participants will be questioned about the number of pills used. The number of tablets taken over a certain time period divided by the total number of tablets prescribed for that time period was used to calculate the non-adherence rates.^16^ If the adherence percentage is 80% or higher, it will be considered adherent; if it is less than 80%, it will be regarded non-adherent. The literature also reports on this threshold. 14,16

Finally, Section-D will be a survey of the post-intervention follow-up to determine the intervention’s acceptability. At the final follow-up interview, participants in the intervention arm will be evaluated for end-user suggestions.

### Statistical Analyses:

Intention-to-treat analysis will be applied. The mean and standard deviation/ median and interquartile ranges will be presented. Frequencies and percentages will be used to depict categorical data. Differences in adherence scores and SBP between two groups (Independent samples t-test/ Mann–Whitney U test) at six months will be applied. For differences in adherence scores and SBP in one group between two time points, a Paired samples t-test /Wilcoxon signed-rank test will be conducted on the data. P < 0.05 will be used as the significance test. Data will be reported as to why it is missing (Table-II). All analysis will be made using SPSS and R-Studio.

**Table-II:** Methods for data analysis

Independent variables (IDVs)	Dependent variables (DVs)	Type of analysis
Categorical (groups)	Continous (SEAMSscore)	(Independent samples t-test/ Paired samples t-test) (Wilcoxon signed-rank test/ Mann–Whitney U test)
Categorical (groups)	Continous (systolic blood pressure)	(Independent samples t-test/ Paired samples t-test) (Wilcoxon signed-rank test/ Mann–Whitney U test)
Categorical (all IDVs)	Categorical (all IDVs)	Chi-squared test

### Consent and Confidentiality:

Participation in the study will be voluntary; trained interviewers will administer informed consent to the participants. Strict confidentiality and privacy will be ensured. Participants will be anonymized in data to protect their identity and password-protected primary servers, mobile devices, and end-to-end encryption WhatsApp; all these measures will be used. Information regarding data security and protection will be provided to participants. There is also a contingency plan to change the intervention strategy for SMS and voice calls in the case of internet connection problems and WhatsApp connectivity or failures.

### Reporting of Adverse Events:

We will document any additional adverse effects relating to participants other than the primary and secondary outcomes.

## DISCUSSION

The current study will assess how successful a comprehensive, multifaceted educational motivation with reminder support as an intervention by using mHealth technology in improving adherence to antihypertensive therapy among hypertensive patients. It will also answer the question of improvement in adherence to medication and control of systolic blood pressure. While some studies have reported improvements in adherence to antihypertensive medications,^14^,^21^ others did not find any improvements in adherence to medication.^22^ Mixed outcomes of the intervention on medication adherence to antihypertensive treatment were revealed by a recent systematic analysis of 23 RCTs (published in 2023). A notable improvement in medication adherence was found in 73.9% of the research, while 26% of the studies did not demonstrate a meaningful influence of the intervention on medication adherence.^11^ In addition, the researcher will analyze the acceptability and efficacy of diverse clinical care aspects using this intervention. A study employed an innovative mHealth designs for CVD patients.^23^ Similarly, new mHealth models have been introduced to improve medication adherence.^24^ Traditionally, written medication schedules, peer counseling,^23^ health education,^25^ counseling,^23^ and routine check-ups have all been utilized to help hypertension patients adhere to their drug regimens.

Compared to other interventions, the current intervention is a comprehensive and novel approach that can be considered an updated version of previous material on the topic because it combines multiple aspects into a single application. According to what we know, this will be the only study to design and test the efficacy of a full, holistic mHealth approach. Furthermore, most intervention studies concentrate on patient adherence to medication rather than blood pressure control.^22^ The current study will gather data on both adherence to medication and as well as SBP, If the intervention can lower the patient’s SBP, it will be a significant improvement in clinical settings with limited resources. There are suggestions for more advanced and effective interventions to improve CVD adherence.^11^

### Limitations:

In the current study, medication adherence will be evaluated through self-reporting. Social desirability bias may lead to an overestimation of actual adherence in self-report questionnaires.

## CONCLUSION

According to this study, if the multifunctional Multi-Aid-Package proves to be a useful mobile health tool for improving hypertension patients’ medication adherence, it will also significantly affect systolic blood pressure. In addition, it will assist the patients in understanding the significance of adhering to their medication routine. The current intervention will be feasible and can easily be employed. In Pakistan and other comparable LMICs, the Multi-Aid-Package ought to be taken into consideration as a means of enhancing adherence to medications among hypertension patients.
